# Rare heterozygous variants in paediatric steroid resistant nephrotic syndrome – a population-based analysis of their significance

**DOI:** 10.1038/s41598-024-68837-2

**Published:** 2024-08-10

**Authors:** C. J. Platt, A. Bierzynska, W. Ding, S. A. Saleem, A. Koziell, M. A. Saleem

**Affiliations:** 1 Bristol Royal Hospital for Children, Bristol, BS2 8NJ UK; 2 Bristol Renal, University of Bristol, Bristol, UK; 3 King’s College, London, UK; 4 King’s College and Evelina, London, UK

**Keywords:** Paediatrics, Kidney, Kidney diseases, Genetics

## Abstract

Genetic testing in nephrotic syndrome may identify heterozygous predicted-pathogenic variants (HPPVs) in autosomal recessive (AR) genes that are known to cause disease in the homozygous or compound heterozygous state. In such cases, it can be difficult to define the variant’s true significance and questions remain about whether a second pathogenic variant has been missed during analysis or whether the variant is an incidental finding. There are now known to be over 70 genes associated with nephrotic syndrome, the majority inherited as an AR trait. Knowledge of whether such HPPVs occur with equal frequency in patients compared to the general population would assist interpretation of their significance. Exome sequencing was performed on 187 Steroid-Resistant Nephrotic Syndrome (SRNS) paediatric patients recruited to a UK rare disease registry plus originating from clinics at Evelina, London. 59 AR podocytopathy linked genes were analysed in each patient and a list of HPPVs created. We compared the frequency of detected HPPVs with a ‘control’ population from the gnomAD database containing exome data from approximately 50,000 individuals. A bespoke filtering process was used for both patients and controls to predict ‘likely pathogenicity’ of variants. In total 130 Caucasian SRNS patients were screened across 59 AR genes and 201 rare heterozygous variants were identified. 17/201 (8.5%) were assigned as ‘likely pathogenic’ (HPPV) using our bespoke filtering method. Comparing each gene in turn, for SRNS patients with a confirmed genetic diagnosis, in 57 of the 59 genes we found no statistically significant difference in the frequency of these HPPVs between patients and controls (In genes *ARHGDIA* and *TP53RK*, we identified a significantly higher number of HPPVs in the control population compared with the patients when filtering was performed with ‘high stringency’ settings only). In the SRNS patients without a genetics diagnosis confirmed, there was no statistically significant difference identified in any gene between patient and control. In children with SRNS, we propose that identification of HPPV in AR podocytopathy linked genes is not necessarily representative of pathogenicity, given that the frequency is similar to that seen in controls for the majority. Whilst this may not exclude the presence of genetic kidney disease, this type of heterozygous variant is unlikely to be causal and each result must be interpreted in its clinical context.

## Introduction

Idiopathic nephrotic syndrome (INS) develops when the renal glomerular filtration barrier is damaged, allowing leakage of albumin into the urine (albuminuria), causing oedema and hypoalbuminaemia. The condition may be categorised according to initial steroid response (steroid sensitive nephrotic syndrome (SSNS) or steroid resistant nephrotic syndrome (SRNS))^1,2^. Most children will have steroid responsive disease with histology showing ‘minimal change’^3^. In patients with SRNS, focal segmental glomerulosclerosis (FSGS) is the most common histological finding. On a global scale, the incidence of FSGS is estimated at 0.2–1.8 cases per 100,000 people per year and contributes significantly to the burden of stage 5 chronic kidney disease (CKD) in children and young people^4^. Around 70% of SRNS patients have no identified mutation in a known podocyte gene (figures reaching as high as 90% in unselected cohorts (NEPTUNE for example), where referral bias is less of a concern) and are thought to have an unidentified ‘circulating factor’ that plays a causative role^5–8^. These patients receive intensive immunosuppression to control their disease but have a poor outcome overall, with relapse after renal transplant seen in up to 60% of cases^9,10^. Improving the molecular stratification of INS will be key to advancing therapeutic strategies and predicting recurrence risk post-transplant for this group of patients^11^.

The advancement of genomic sequencing technologies has identified the next largest subset of SRNS as being caused by single gene (monogenic) disorders, which account for up to 30% of cases^12–14^. Monogenic SRNS is associated with a more aggressive renal decline and little response to intensified immunosuppression. In addition, monogenic patients do not tend to have disease recurrence post-transplant^9^.

There are currently around 70 genes, mostly with AR inheritance, associated with monogenic SRNS^9,14–16^. Targeted gene panels have been developed based on this knowledge, facilitating rapid turnaround of results for affected patients^16^, with paediatric SRNS patients in the UK now recommended to have genetic testing by next generation sequencing (NGS)^17^. The ‘proteinuric renal disease’ panel covers 55 ‘green’ genes as outlined in the National Test Directory centrally commissioned by NHS England^18,19^. Rare variants in the ‘green’ or definitive SRNS genes are assessed for their significance using standard guidelines developed by the American College of Medical Genetics (ACMG)^20^. However, single rare heterozygous predicted-pathogenic variants (HPPVs) within AR genes that create uncertainty may be identified^21^, a problem recognised by Nephrologists. In such situations, the possibility that current sequencing technology is not able to reliably detect a second variant where one exists, or alternatively that a second variant resides either within the non-coding genome or in another podocytopathy gene, may lead to an assumption about pathogenicity.

A typical human genome contains 5 million variants of which 40,000 to 200,000 are designated ‘rare’ (MAF < 0.5%)^22^. Data from an exome-based study from Europe also shows that individuals carry at least 2 pathogenic variants in autosomal recessive (AR) genes^23^. This estimate can be used to identify couples at risk of having a child with an AR disease. Identification of an HPPV in a nephrotic gene, may therefore be incidental.

Another recent large-scale exome study involving sporadic and familial cases of FSGS in 662 individuals, showed that the there was a non-trivial rate of apparently ‘disease-causing’ (loss of function, non-synonymous) rare variants in known FSGS genes in healthy controls^24^.

There have been over 5000 diseases/traits found to have a monogenic cause^25^. Understanding the relevance of finding potentially pathogenic rare variants from the genomes of affected patients may involve a comparison with the frequency/distribution of similar variants in a control population^26^. A study in patients with Joubert’s syndrome (ciliopathy) for example, where predicted pathogenic variants can be detected in 2 or more Joubert-associated genes, used the comparison with an ExAC population database ‘control’ group to conclude that the distribution of variants within diseased individuals and healthy individuals was similar, and therefore not supporting the role for digenic inheritance in the condition^27^.

Continuing within the spectrum of genetic renal conditions, the study of rare variants has yielded information about new gene-disease associations and modifiers, in chronic kidney disease (CKD) for example. A comparison of the exome data on 3150 patients with chronic kidney disease (CKD) and 9563 healthy controls, observed an increased rare variant burden in several genes previously not associated with CKD (*CPT2* for example)^28^. In the recessive salt-losing tubulopathy, Gitleman’s syndrome, 10% of patients are identified as having only one pathogenic variant in *SLC12A3* using current sequencing methodologies. Long read sequencing of the gene in 67 such cases detected a second pathogenic/likely pathogenic variant in 45 (67%) of these cases^29^. This may be an additional tool to consider for patients with SRNS in similar circumstances.

There is increasing evidence that there is a spectrum of ‘recessiveness’ in heterozygous carriers of traditionally recessive genes. For some conditions, it has become apparent that there is a phenotype in carriers that is often milder, while for other conditions, it appears that they are properly recessive^30–34^. Two studies from the Finnish Biobank (FinnGen) suggest that there are HPPV effects in patients with mono-allelic *NPHS1* (one of the commonest genes implicated in childhood nephrotic syndrome) variants^31,32,34,35^. Our study was not designed to assess such mild phenotypic HPPV effects and the absence of reporting such effects does not exclude the possibility of their relevance in paediatric SRNS.

For this study, we hypothesised that in paediatric SRNS, the frequency of HPPVs in recessive podocytopathy linked genes (Supplementary Table 1) would be similar to the frequency seen in a large European cohort from the gnomAD population database.

## Methods

**Figure 1:**
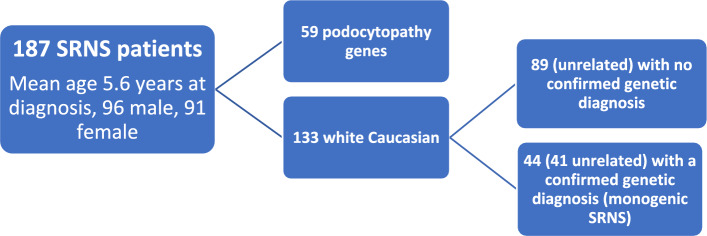
The UK Paediatric SRNS cohort.

The study population was drawn from the UK Registry for Rare Kidney Disease database (RADAR; www.ukkidney.org), a national registry into which patients with SRNS are recruited^33^ as well as Evelina London, Guys and St Thomas’ Hospital Trust. All data usage and analysis were performed in accordance with the regulations and guidelines from RADAR and the University of Bristol. The details of this study cohort (Fig. 1) have previously been described, and the report includes details of the precise pathogenic variants in its supplementary pages^9^. From 187 patients with SRNS, 133 were of white Caucasian ethnicity. Of the 44 confirmed to have monogenic SRNS, 3 had an identical twin sibling and were therefore excluded to leave only 1 of each twin pair in the cohort. This left 130 white Caucasian patients for the analysis, 41 with a confirmed genetic diagnosis (‘monogenic SRNS’) and 89 in whom a genetic diagnosis had not been identified.

Our control population was drawn from the European (Non-Finnish) population in the Genome Aggregation Database (gnomAD (http://gnomad.broadinstitute.org version 2.1.1)^34^.

Exome data from 59 AR ‘nephrotic’ genes was systematically filtered to leave a list of rare (MAF < 0.01) heterozygous variants in the patient and control populations for comparison. 47 genes in the non-monogenic patients, and 28 genes in the monogenic patients were identified to contain at least 1 rare heterozygous variant in an AR podocytopathy gene.

A series of further filtering steps (outlined below) were employed to compile a final list of HPPVs in patients and controls (of note, this filtering was not designed to identify causative ‘mutations’ as might occur in a clinical analysis). For the patients already confirmed as monogenic (causative mutation in a podocytopathy linked gene identified previously), the causative pathogenic variants were filtered out of the data prior to comparison of remaining variants with the control group.

The GnomAD database does not provide data *per individual*, and therefore comparisons of variant frequencies were performed for the group as a whole.

Four final groups were established for comparison as follows: Rare heterozygous variants (independent of pathogenicity predictions) in confirmed monogenic patients vs controls/rare heterozygous variants (independent of pathogenicity predictions) in patients with no genetic diagnosis identified vs controls/HPPVs in monogenic patients vs controls/HPPVs in non-monogenic patients vs controls.

Step 1 (filtering):

- Remove intronic variants (outside ± 10 base pairs from intron/exon junction).
- Remove variants seen as homozygous.
- Remove variants with MAF > 0.01.

Step 2 (variants must meet the following criteria):

- Variant likely to disrupt the splice site (± 2 base pairs from intron/exon junction).
- Variant was an insertion/deletion (frameshift/non-frameshift).
- Variant resulted in a gain or loss of a stop codon.
- Variant was missense and predicted to be pathogenic by in-silico tools (high and low stringency settings were applied for comparison*).

*Low stringency = any 4 of the 6 in-silico tools used agreed.

*High stringency = CADD and REVEL and 3 out of 4 remaining in-silico tools agreed.

Step 3 (pathogenicity predictions):

- The Ensembl Variant Effect Predictor (VEP) was used to assign pathogenicity predictions to the list of remaining variants. A set of 6 in-silico prediction tools were used (CADD^36^/Revel^35^/MutPred^37^/Fathmm-XL^38^/Polyphen^39^/SIFT^40^).

Taking each gene in turn, the total number of (a) rare heterozygous variants and (b) HPPVs, along with corresponding allele counts (AC) and allele numbers (AN), were recorded for both patients and controls. For the control population, the number of individuals was estimated by taking an average of the ANs recorded in gnomAD for each variant and dividing it by 2 (which is why the number of individuals (denominator in Table 1) in the control population varies).

**Table 1:** Results summary showing number of variants per patient/control per gene for monogenic and non-monogenic SRNS.

	Confirmed monogenic SRNS		P-value	SRNS (no genetic diagnosis confirmed)		P-value
	Patients	gnomAD		Patients	gnomAD	
Rare heterozygous variants per patient per gene	0.018 (43/41)	0.038 (125,083/55,773)	0.4992	0.030 (158/89)	0.038 (125,083/55,773)	0.7003
Likely pathogenic het (low stringency analysis) per patient per gene	0.003 (8/41)	0.009 (28,361/55,844)	0.7163	0.006 (30/89)	0.009 (28,361/55,844)	0.7690
Likely pathogenic het (high stringency analysis) per patient per gene	0.002 (4/41)	0.004 (13,108/55,796)	0.8209	0.003 (13/89)	0.004 (13,108/55,796)	0.8374

Summary of results showing average number of variants per patient per gene compared with average number of variants per individual per gene in the control group (gnomAD) in the different categories (numbers in brackets beneath represent the actual number of variants divided by the number of patients/number of individuals). For example, in confirmed monogenic patients, 43 rare heterozygous variants were identified in 41 individuals across 59 AR nephrotic genes compared with 125,083 rare heterozygous variants in 55,773 individuals from the gnomAD population.

### Statistical analysis

The hypothesis that the allele count (AC) in the patient and control groups are statistically consistent (null hypothesis) was tested by calculating the Chi-squared (${\chi }^{2} )$ statistic. In the present study the ${\chi }^{2}$ statistic was computed including the Yates’ correction, which is considered appropriate given the relatively small sample size of the patient group. A Bonferroni correction was applied to adjust the p-values considering the multiple (59 genes) comparisons. For comparisons in Table 1 we applied sampling theory for the proportions to calculate p values (comparing variants per patient/control per gene).

### Ethics approval

South West-Central Bristol Ethics committee (09/H0106/80).

Our cohort of paediatric SRNS cases was recruited via the United Kingdom Registry for Rare Kidney Diseases (RaDaR) and included all tertiary paediatric nephrology centres in the UK. Detailed phenotypic information was entered online (https://www.radar.nhs.uk/), and laboratory data were automatically populated via links to the UK Renal Registry (www.renalreg.org). Appropriate informed consent from parents and/or carers was collected, and assent for collection of data and genetic analysis obtained. The study was approved by the South West research ethics committee and the institutional review board at each recruiting centre.

## Results

Rare heterozygous variants (independent of pathogenicity predictions) were identified in 47 genes from the patients with no genetic diagnosis identified and in 28 genes from the confirmed monogenic patients (Supplementary Tables 2, 3). HPPVs were identified in 10 genes from the group with no genetic diagnosis and in 3 genes from the confirmed monogenic patients (Tables 2, 3), using a high-stringency in-silico approach. HPPVs were identified in 17 genes from the patients with no genetic diagnosis and in 7 genes from the confirmed monogenic patients (Supplementary Tables 4, 5), using a low-stringency in-silico approach. Table 1 provides a summary. The denominator for the gnomAD group changes because the allele number (AN) in gnomAD is different for each variant (as explained above).

**Table 2:** Heterozygous predicted-pathogenic variants (HPPVs) in patients with no confirmed genetic diagnosis (N = 89) versus controls using high stringency prediction tools (as defined in text).

Gene	Accession#	gnomAD		Patients (no genetic diagnosis confirmed) n = 89		Chi-2	P-value	P-value with Bonferroni correction
		AC (HET)	AN	AC (HET)	AN			
*ADCK4*	NM_024876	318	113,427	0	178	0.000006	0.998020	1
*ALG1*	NM_019109	68	111,491	0	178	1.417967	0.233738	1
*ANKFY1*	NM_001257999	107	109,447	0	178	0.614285	0.433179	1
*ARHGDIA*	NM_001185077.1	24	100,432	0	178	4.940156	**0.026240**	1
*AVIL*	NM_006576	242	116,358	0	178	0.046142	0.829919	1
*CD151*	NM_004357	48	113,350	0	178	2.401900	0.121188	1
*CD2AP*	NM_012120	65	116,511	0	178	1.623898	0.202549	1
*CDK20*	NM_001039803.2	71	109,054	0	178	1.279398	0.258011	1
*CFH*	NM_000186	94	112,950	0	178	0.840215	0.359335	1
*COL4A3*	NM_000091	978	111,848	0	178	0.722285	0.395396	1
*COL4A4*	NM_000092	478	114,113	0	178	0.080730	0.776311	1
*COQ2*	NM_001358921.1	117	100,202	0	178	0.413668	0.520113	1
*COQ6*	NM_182480.2	83	113,890	0	178	1.062164	0.302722	1
*CRB2*	NM_173689	401	99,883	0	178	0.064176	0.800013	1
*CUBN*	NM_001081	802	115,691	2	178	0.057291	0.810830	1
*DGKE*	NM_003647	72	112,193	0	178	1.308972	0.252581	1
*DLC1*	NM_182643	149	114,216	0	178	0.311028	0.577050	1
*EMP2*	NM_001424	120	114,747	1	178	0.522748	0.469672	1
*FAT1*	NM_005245	973	113,157	1	178	0.000584	0.980724	1
*GAPVD1*	NM_015635	117	112,265	0	178	0.536410	0.463924	1
*ITGA3*	NM_002204	70	106,037	0	178	1.251343	0.263296	1
*ITGB4*	NM_000213	744	113,118	0	178	0.385915	0.534455	1
*ITSN1*	NM_001001132	101	108,076	0	178	0.673177	0.411946	1
*ITSN2*	NM_006277	263	113,682	0	178	0.019274	0.889585	1
*KANK1*	NM_001256877	484	116,015	1	178	0.079923	0.777402	1
*KANK2*	NM_001136191	109	113,511	0	178	0.637178	0.424735	1
*KANK4*	NM_181712	71	116,215	0	178	1.414042	0.234387	1
*KIRREL1*	NM_018240	35	97,632	0	178	2.995183	0.083512	1
*LAMA5*	NM_005560	105	104,615	0	178	0.581647	0.445668	1
*LAMB2*	NM_002292	558	114,928	0	178	0.153596	0.695123	1
*LCAT*	NM_000229.2	139	110,740	0	178	0.344795	0.557074	1
*MAGI2*	NM_012301	79	109,232	0	178	1.076186	0.299552	1
*MMACHC*	NM_015506	619	115,776	0	178	0.214794	0.643036	1
*MYO1E*	NM_004998	399	109,728	0	178	0.033254	0.855304	1
*NEU1*	NM_000434.4	96	112,969	0	178	0.808345	0.368610	1
*NPHS1*	NM_004646	261	112,349	1	178	0.017732	0.894067	1
*NPHS2*	NM_014625	251	112,669	1	178	0.026535	0.870600	1
*NPHP4*	NM_015102	579	111,845	2	178	0.362854	0.546925	1
*NUP107*	NM_020401	117	113,713	0	178	0.551463	0.457720	1
*NUP133*	NM_018230	75	113,836	0	178	1.254967	0.262606	1
*NUP160*	NM_015231	176	114,630	0	178	0.189632	0.663223	1
*NUP205*	NM_015135	96	113,817	0	178	0.819629	0.365289	1
*NUP85*	NM_024844.5	93	114,662	0	178	0.880544	0.348053	1
*NUP93*	NM_014669.5	53	110,351	0	178	2.018566	0.155386	1
*OSGEP*	NM_017807	48	114,814	0	178	2.443811	0.117989	1
*PDSS2*	NM_020381	66	112,171	0	178	1.498632	0.220882	1
*PLCe1*	NM_016341	145	113,950	0	178	0.332554	0.564159	1
*PMM2*	NM_000303	339	110,869	2	178	1.670773	0.196155	1
*PTPRO*	NM_030667	94	115,363	0	178	0.873220	0.350065	1
*SCARB2*	NM_005506	94	115,251	0	178	0.871678	0.350491	1
*SGPL1*	NM_003901	31	112,495	1	178	4.003282	**0.045412**	1
*SMARCAL1*	NM_014140	106	112,806	0	178	0.665702	0.414554	1
*TNS2*	NM_015319	369	113,011	0	178	0.011162	0.915861	1
*TP53RK*	NM_033550.4	19	104,994	0	178	6.819325	**0.009018**	0.532062
*TPRKB*	NM_001330386	36	104,462	0	178	3.150080	0.075923	1
*TTC21B*	NM_024753	438	115,293	0	178	0.045696	0.830728	1
*WDR73*	NM_032856	159	106,387	0	178	0.207558	0.648688	1
*XPO5*	NM_020750	51	111,923	0	178	2.172586	0.140490	1
*ZMPSTE24*	NM_005857	213	114,814	1	178	0.086251	0.768999	1

Control population from gnomAD European non-Finnish.

*AC* allele count, *AN* allele number.

P < 0.05 highlighted in bold.

**Table 3:** Heterozygous predicted-pathogenic variants (HPPVs) in confirmed monogenic patients (N = 41) versus controls using high stringency prediction tools (as defined in text).

Gene	Accession#	gnomAD		Confirmed monogenic patients (n = 41)		Chi-2	P-value	P-value with Bonferroni correction
		AC (HET)	AN	AC (HET)	AN			
*ADCK4*	NM_024876	318	113,427	0	82	0.319102	0.572148	1
*ALG1*	NM_019109	68	111,491	0	82	4.057817	0.043967	1
*ANKFY1*	NM_001257999	107	109,447	0	82	2.204751	0.137586	1
***ARHGDIA***	**NM_001185077.1**	**24**	**100,432**	**0**	**82**	**11.800615**	**0.000592**	**0.034928**
*AVIL*	NM_006576	242	116,358	0	82	0.639138	0.424023	1
*CD151*	NM_004357	48	113,350	0	82	6.246629	**0.012443**	0.734137
*CD2AP*	NM_012120	65	116,511	0	82	4.520117	0.033499	1
*CDK20*	NM_001039803.2	71	109,054	0	82	3.744956	0.052967	1
*CFH*	NM_000186	94	112,950	0	82	2.738513	0.097956	1
*COL4A3*	NM_000091	978	111,848	0	82	0.066035	0.797200	1
*COL4A4*	NM_000092	478	114,113	0	82	0.071950	0.788518	1
*COQ2*	NM_001358921.1	117	100,202	0	82	1.712255	0.190693	1
*COQ6*	NM_182480.2	83	113,890	0	82	3.250876	0.071385	1
*CRB2*	NM_173689	401	99,883	0	82	0.089396	0.764946	1
*CUBN*	NM_001081	802	115,691	2	82	1.532295	0.215768	1
*DGKE*	NM_003647	72	112,193	0	82	3.812014	0.050886	1
*DLC1*	NM_182643	149	114,216	0	82	1.448538	0.228763	1
*EMP2*	NM_001424	120	114,747	0	82	2.006625	0.156613	1
*FAT1*	NM_005245	973	113,157	0	82	0.059960	0.806559	1
*GAPVD1*	NM_015635	117	112,265	0	82	2.016495	0.155598	1
*ITGA3*	NM_002204	70	106,037	0	82	3.681256	0.055027	1
*ITGB4*	NM_000213	744	113,118	0	82	0.002834	0.957543	1
*ITSN1*	NM_001001132	101	108,076	0	82	2.345398	0.125653	1
*ITSN2*	NM_006277	263	113,682	0	82	0.509904	0.475180	1
*KANK1*	NM_001256877	484	116,015	1	82	0.072716	0.787423	1
*KANK2*	NM_001136191	109	113,511	0	82	2.259728	0.132777	1
*KANK4*	NM_181712	71	116,215	0	82	4.049239	0.044191	1
*KIRREL1*	NM_018240	35	97,632	0	82	7.550098	**0.006001**	0.354059
*LAMA5*	NM_005560	105	104,615	1	82	2.098090	0.147483	1
*LAMB2*	NM_002292	558	114,928	0	82	0.026378	0.870981	1
*LCAT*	NM_000229.2	139	110,740	0	82	1.536643	0.215118	1
*MAGI2*	NM_012301	79	109,232	0	82	3.282752	0.070011	1
*MMACHC*	NM_015506	619	115,776	0	82	0.008797	0.925272	1
*MYO1E*	NM_004998	399	109,728	0	82	0.137619	0.710660	1
*NEU1*	NM_000434.4	96	112,969	0	82	2.664128	0.102634	1
*NPHS1*	NM_004646	261	112,349	0	82	0.505221	0.477215	1
*NPHS2*	NM_014625	251	112,669	0	82	0.553712	0.456805	1
*NPHP4*	NM_015102	579	111,845	0	82	0.013630	0.907060	1
*NUP107*	NM_020401	117	113,713	0	82	2.053172	0.151889	1
*NUP133*	NM_018230	75	113,836	0	82	3.689880	0.054744	1
*NUP160*	NM_015231	176	114,630	0	82	1.115422	0.290906	1
*NUP205*	NM_015135	96	113,817	0	82	2.690524	0.100947	1
*NUP85*	NM_024844.5	93	114,662	0	82	2.832373	0.092382	1
*NUP93*	NM_014669.5	53	110,351	0	82	5.398492	0.020154	1
*OSGEP*	NM_017807	48	114,814	0	82	6.339210	**0.011810**	0.69679
*PDSS2*	NM_020381	66	112,171	0	82	4.239177	0.039501	1
*PLCe1*	NM_016341	145	113,950	0	82	1.504910	0.219917	1
*PMM2*	NM_000303	339	110,869	0	82	0.249321	0.617554	1
*PTPRO*	NM_030667	94	115,363	0	82	2.815390	0.093364	1
*SCARB2*	NM_005506	94	115,251	0	82	2.811803	0.093573	1
*SGPL1*	NM_003901	31	112,495	0	82	10.104416	**0.001479**	0.087261
*SMARCAL1*	NM_014140	106	112,806	0	82	2.327766	0.127084	1
*TNS2*	NM_015319	369	113,011	0	82	0.202765	0.652498	1
***TP53RK***	**NM_033550.4**	**19**	**104,994**	**0**	**82**	**15.890826**	**0.000067**	**0.003953**
*TPRKB*	NM_001330386	36	104,462	0	82	7.890809	0.004969	0.293171
*TTC21B*	NM_024753	438	115,293	0	82	0.114904	0.734628	1
*WDR73*	NM_032856	159	106,387	0	82	1.166604	0.280100	1
*XPO5*	NM_020750	51	111,923	0	82	5.739829	**0.016584**	0.978456
*ZMPSTE24*	NM_005857	213	114,814	0	82	0.798632	0.371503	1

Control population from gnomAD European (non-Finnish).

*AC* allele count, *AN* allele number.

P < 0.05 highlighted in bold.

In *MMACHC (*Metabolism of Cobalamin Associated C), we observed significantly more rare heterozygous variants (independent of pathogenicity predictions) in patients with no genetic diagnosis identified versus controls (9/89 versus 1355/58,114 (corrected P = 0.00059)). This difference was not observed in the confirmed monogenic patients (*MMACHC* corrected p value for comparison of monogenic vs control = 1) nor was it observed when variants were filtered using in-silico pathogenicity predictions.

### Patients without a genetic diagnosis

158 rare heterozygous variants were found in 59 recessive genes from 89 unrelated patients. 125,083 rare heterozygous variants in the same 59 genes were identified in 55,773 individuals from gnomAD (Table 1).

Using a low stringency in-silico approach (Supplementary Table 4), 30 HPPVs (19% of the original 158) were found in 59 genes from the 89 patients without a genetic diagnosis compared with 28,361 in the same 59 genes from 55,844 individuals in gnomAD (controls). When the high stringency approach (Table 2) was used, 13 variants (8.2% of the original 158) were found in 59 genes in the patients, versus 13,108 in 55,796 individuals from gnomAD (controls). Comparing each gene in turn, the number of these HPPVs was not significantly different in patients versus controls.

### Confirmed monogenic patients

43 rare heterozygous variants were found in 59 genes from 41 unrelated genetic patients compared with 125,083 in 55,773 individuals in gnomAD (controls) (Table 3).

Using a low stringency in-silico approach (Supplementary Table 5), 8 HPPVs (18.6% of the original 43) were found in 59 genes from 41 patients, compared with 28,361 variants in 55,844 individuals in gnomAD (controls). With a high stringency approach (Table 3), 4 HPPVs (9.3% of the original 43) were found in 59 genes from 41 patients compared with 13,108 variants in 55,796 individuals in gnomAD (controls).

In the *ARHGDIA* (Rho GDP Dissociation Inhibitor Alpha) and *TP53RK* (TP53 Regulating Kinase) genes, we observed a significantly higher number of HPPVs in the control population compared with patients (24/50,216 vs. 0/41 (corrected P = 0.034928) and 19/52,497 vs 0/41 (corrected P = 0.003953) respectively, Table 3) using the high stringency approach.

Comparing the remaining 57 genes in turn, the number of HPPVs were not significantly different in patients versus gnomAD controls.

## Discussion

Whole exome sequencing (WES) in SRNS has been shown to be a helpful tool for diagnosis and also for identification of novel candidate genes^41^. This is also true in other renal phenotypes, for example in cohorts of children with increased renal echogenicity and chronic kidney disease, WES has been shown to identify a causative mutation in up to 63% of cases^42^.

Childhood onset SRNS is a disease that makes a large contribution to the burden of end stage renal disease seen in paediatrics. Based on our current understanding, a monogenic cause is identified in the minority of cases and there remains uncertainty about missing genetic data in the remainder^43,44^. The relevance of finding rare heterozygous variants in recessive podocytopathy-linked genes in children with SRNS is currently unknown. Our data has shown that there was no statistically significant difference in the prevalence of rare heterozygous variants identified in AR podocytopathy-linked genes between patients and controls for all but one gene (*MMACHC* in the patients with no confirmed genetic diagnosis), and when pathogenicity predictions were applied to these rare heterozygous variants, so that only those variants assigned ‘likely pathogenic’ (HPPV) remained, the prevalence was statistically significantly higher in the control population versus patients in the ‘confirmed monogenic SRNS’ group in only 2 genes (*ARGHDIA* and *TP53RK*), which we suggest may because we had removed all ‘causative mutations’ in the ‘confirmed monogenic’ patient group prior to comparing the frequencies of HPPV.

Mutations in the *MMACHC* (metabolism of Cobalamin Associated C) gene are responsible for Cobalamin C deficiency^45^. Renal manifestations of Cobalamin C defects include thrombotic microangiopathy, tubulointerstitial nephritis, proximal renal tubular acidosis and nephrotic syndrome^45^. We see a higher prevalence (corrected P = 0.00059) of rare heterozygous variants (independent of pathogenicity predictions) in *MMACHC* in the patients with SRNS in whom a genetic diagnosis has not been confirmed (Supplementary Table 2). This may be because these patients have been wrongly assigned as ‘non-genetic’ and a second unidentified pathogenic variant resides elsewhere in the genome, or, that these variants are relevant via some other mechanism—for example, through their ability to change/modify the expression of other genes involved in the production of a ‘circulating factor’ for example.

The ‘modifier’ concept has previously been described in association with renal disease progression relating to *NPHS2* nephrotic syndrome^46^ and more recently, in an Indian cohort of paediatric SRNS, where a potential role for collagen gene modifiers was suggested, noting the high incidence of *COL4A* variants identified in their patients^47^.

This statistically different result in *MMACHC* is not seen after pathogenicity predictions are applied to the analysis and we find comparable results for the prevalence of predicted pathogenic variants (HPPV) in both monogenic and ‘genetic diagnosis not confirmed’ SRNS groups for this gene.

There were significantly more HPPVs (identified using prediction tools at high stringency) in controls compared with ‘confirmed monogenic’ SRNS patients in *ARGHDIA* and *TP53RK.* These genes are the smallest of the group analysed (*ARHGDIA* = 3658 bases and *TP53RK* = 5080 bases) and the number of HPPVs correspondingly small also in the gnomAD data set (24 and 19 respectively). There were no HPPVs identified in any of the patients for these genes. The gene size compounds the effects of the small sample size for this sub-group of patients. This significant difference is not evident in the ’no genetic diagnosis confirmed’ patients (n = 89), nor when prediction tools were applied to the filtering with a lower level of stringency.

In a mouse model of acute kidney injury, rare variants in the *TNFAIP3* (TNF alpha-induced protein 3) gene were identified as having anti-inflammatory effects, and as such they were deemed ‘protective’^48^. The potential to use the understanding of the mechanisms through which such protective variants may exert their effects can be used in drug development, and as such is of high importance.

Comparing rare heterozygous variants in AR genes between patients and general population databases to determine their significance has been done before. For example, in Parkinson’s disease, the recessive gene *PINK1* is associated with monogenic forms of the condition. A study involving 1100 patients with Parkinson’s disease demonstrated that rare heterozygous variants in *PINK1* were seen at an equal frequency in patients (1.8%) and healthy controls (1.5%), with the conclusion that these rare variants are likely to play only a minor susceptibility role in the context of a multifactorial disease model^49,50^. Perhaps rare variants in the *MMACHC* gene act by increasing the ‘susceptibility’ to SRNS.

Our understanding of ‘recessiveness’ in disease is also expanding such that we are now aware that the presence of rare heterozygous variants in recessive genes may be associated with a phenotype in certain conditions^51^. For example, in the case of the collagen genes, where patients with rare heterozygous variants in *COL4A3/COL4A4* often have persistent microscopic haematuria. The rate of progression to end stage renal disease is much lower in patients harbouring a single pathogenic variant in the recessive *COL4A4* gene when compared with those found to have bi-allelic *COL4A4* pathogenic variants^52,53^.

While there are likely to be several mechanisms that contribute to the expression of variants, there are cis-regulatory elements and eQTLs (expression quantitative trait locus) that control transcription, playing a role in transcription from the relevant allele^54^. In the case of a single pathogenic variant in a traditionally recessive disease, the regulation of whether the wild type or potentially pathogenic rare variant is expressed might contribute to pathogenicity. The control of transcription by eQTLs is cell type dependent, and we are only just beginning to have the tools to map these in the kidney^55^. We do not have this data for the patients in this study, and although there are similar numbers of HPPVs in our cases and controls, it is possible that other elements controlling whether these pathogenic variants are expressed could contribute to the phenotype.

The presence of a rare variant per se*,* does not predict disease and our data indicates that in common with other rare diseases, rare variants are found throughout the genome in disease-related genes from healthy individuals^53,56^. We see examples of this from other studies, for example, in an exome sequencing case–control study of 363 patients with familial and sporadic FSGS, 5.5% of the control group were found to have a rare variant that would have been deemed ‘causal’ if found in a patient^24^. A large exome analysis of 7974 healthy adults was assessed for the presence of pathogenic variants in 626 genes known to be associated with mendelian kidney and urinary tract disorders found, after stringent filtering, that 1.4% of them had a candidate pathogenic variant^57^.

Interpreting the significance of heterozygous variants that are predicted to be pathogenic (HPPV) in recessive disorders is challenging. We have approached this in a comprehensively phenotyped and genotyped cohort of paediatric patients with SRNS, thus ruling out incorrect phenotype being an issue. In the case of heterozygous changes in AR genes which are of unknown significance, other mechanisms to consider to avoid misinterpretation, are whether a second pathogenic variant resides within the non-coding region, or within the exome, but is missed due to the constraints of existing sequencing technology. A second pathogenic variant could also be present in a novel SRNS gene or possibly part of digenic inheritance including a novel SRNS gene^58,59^. However, the majority of gene-negative patients from this cohort were also subsequently whole genome sequenced, and this did not increase the rate of detection of mutations within the non-coding space^60^. Equally with advances in NGS technology and good coverage across all genes tested, false negatives, unless residing in hidden parts of the genome and requiring long read sequencing, become less of an issue. Our study therefore suggests that these possibilities will in practice only be relevant for a minority of cases, and as such, in the absence of evidence for other genetic mechanisms, heterozygous changes in AR podocyte genes should be reported as incidental rather than causal.

We propose that this study serves as a pilot for future larger scale analyses and that such analyses include additional sub-groups (circulating factor disease, patients responding to Rituximab etc.), where identification of enriched rare heterozygous variants in specific genes from patients may help to define new and important molecular pathways.

### Limitations

There are a number of limitations to this study which are important to address. The sample size is relatively small, even if deeply phenotyped. The generalisability of the findings is limited to people of European ancestry. It was also not possible to assess the true number of HPPVs per individual in controls, as the data was only available for the population. Finally, our assessment of ‘predicted pathogenicity’ was rather more basic than that used for clinical reporting (we used a modified version of the standard ACMG criteria for variant classification), and it is possible therefore, that some of the variants were mis-assigned.

## Conclusions

This study set out to investigate a previously unanswered question about the relevance of rare predicted-pathogenic heterozygous variants in recessive ‘podocytopathy-linked’ genes in a UK paediatric SRNS cohort. We conclude, whilst numbers were limited, there appears to be no significant difference in the number of HPPVs detected in paediatric SRNS compared with ancestrally-matched controls in the patients with no confirmed genetic diagnosis and for the majority of genes (57/59) in the confirmed monogenic patients. Despite limitations and the possibility of missed pathogenic changes in coding/non-coding regions as well as novel genes, our data provides evidence to support the observation that HPPVs are infrequently causal and associated with a pathogenic genotype in children with SRNS. This should reassure clinicians, and emphasises that whilst genetics may be a powerful tool to establish a diagnosis, clinical interpretation of less definitive results should be more cautious and may not provide the answer. One caveat does however remain for the interpretation of UK SRNS Panel testing through the National test directory. Since only 55 of a possible 70 genes linked to podocytopathy satisfy the criteria of being a ‘green gene’ or one with incontrovertible proof of pathogenicity, a negative result may not necessarily exclude the presence of genetic disease and in this instance the clinical presentation may be the more accurate.

### Supplementary Information

Supplementary Tables.

## Data Availability

The datasets generated and analysed during the current study will be made available upon request from the corresponding author. Upon acceptance of the paper for publication they will be made available in an ISDN approved repository.
