# Supplementary material for: Rare heterozygous variants in paediatric steroid resistant nephrotic syndrome – a population-based analysis of their significance
Source: Sci Rep. 2024 Aug 10;14:18568. doi: 10.1038/s41598-024-68837-2 (PMC11316802; doi:10.1038/s41598-024-68837-2)
Supplement: Supplementary file 1 — Supplementary Tables. [file 41598_2024_68837_MOESM1_ESM.docx]

**Supplementary Table 1**: List of 59 autosomal recessive genes

| *ADCK4* |
| --- |
| *ALG1* |
| *ANKFY1* |
| *ARHGDIA* |
| *AVIL* |
| *CD151* |
| *CD2AP* |
| *CDK20* |
| *CFH* |
| *COL4A3* |
| *COL4A4* |
| *COQ2* |
| *COQ6* |
| *CRB2* |
| *CUBN* |
| *DGKE* |
| *DLC1* |
| *EMP2* |
| *FAT1* |
| *GAPVD1* |
| *ITGA3* |
| *ITGB4* |
| *ITSN1* |
| *ITSN2* |
| *KANK1* |
| *KANK2* |
| *KANK4* |
| *KIRREL1* |
| *LAMA5* |
| *LAMB2* |
| *LCAT* |
| *MAGI2* |
| *MMACHC* |
| *MYO1E* |
| *NEU1* |
| *NPHS1* |
| *NPHS2* |
| *NPHP4* |
| *NUP107* |
| *NUP133* |
| *NUP160* |
| *NUP205* |
| *NUP85* |
| *NUP93* |
| *OSGEP* |
| *PDSS2* |
| *PLCe1* |
| *PMM2* |
| *PTPRO* |
| *SCARB2* |
| *SGPL1* |
| *SMARCAL1* |
| *TNS2* |
| *TP53RK* |
| *TPRKB* |
| *TTC21B* |
| *WDR73* |
| *XPO5* |
| *ZMPSTE24* |

| **Supplementary Table 2**: Comparison of rare heterozygous variants (independent of pathogenicity predictions) in gnomAD and non-monogenic SRNS patients | | | | | | | |  |
| --- | --- | --- | --- | --- | --- | --- | --- | --- |
|  |  | **gnomAD** | | **Patients (no genetic diagnosis confirmed n=89)** | |  |  |  |
| Gene | Accession # | AC (HET) | AN | AC (HET) | AN | Chi-2 | p-value | p-value with Bonferroni correction |
| *ADCK4* | NM_024876 | 943 | 114018 | 1 | 178 | 0.000560 | 0.981120 | 1 |
| *ALG1* | NM_019109 | 1284 | 110804 | 2 | 178 | 0.094010 | 0.759140 | 1 |
| *ANKFY1* | NM_001257999 | 1720 | 111605 | 3 | 178 | 0.022010 | 0.882060 | 1 |
| *ARHGDIA* | NM_001185077.1 | 368 | 86011 | 0 | 178 | 0.089516 | 0.764793 | 1 |
| *AVIL* | NM_006576 | 1495 | 115537 | 3 | 178 | 0.016860 | 0.896680 | 1 |
| *CD151* | NM_004357 | 528 | 115000 | 1 | 178 | 0.124090 | 0.724640 | 1 |
| *CD2AP* | NM_012120 | 991 | 115634 | 1 | 178 | 0.000400 | 0.983980 | 1 |
| *CDK20* | NM_001039803.2 | 732 | 109062 | 0 | 178 | 0.405725 | 0.524147 | 1 |
| *CFH* | NM_000186 | 1543 | 114823 | 2 | 178 | 0.005010 | 0.943580 | 1 |
| *COL4A3* | NM_000091 | 2801 | 112570 | 3 | 178 | 0.199300 | 0.655290 | 1 |
| *COL4A4* | NM_000092 | 2359 | 115038 | 3 | 178 | 0.006230 | 0.937090 | 1 |
| *COQ2* | NM_001358921.1 | 654 | 89426 | 0 | 178 | 0.496212 | 0.481169 | 1 |
| *COQ6* | NM_182480.2 | 1176 | 111596 | 1 | 178 | 0.075690 | 0.783230 | 1 |
| *CRB2* | NM_173689 | 2198 | 99200 | 3 | 178 | 0.050840 | 0.821610 | 1 |
| *CUBN* | NM_001081 | 7434 | 116016 | 13 | 178 | 0.111810 | 0.738090 | 1 |
| *DGKE* | NM_003647 | 1297 | 112590 | 4 | 178 | 1.032310 | 0.309620 | 1 |
| *DLC1* | NM_182643 | 3586 | 114447 | 0 | 178 | 4.769959 | 0.028961 | 1 |
| *EMP2* | NM_001424 | 635 | 116385 | 1 | 178 | 0.230230 | 0.631350 | 1 |
| *FAT1* | NM_005245 | 9183 | 115311 | 11 | 178 | 0.547640 | 0.459280 | 1 |
| *GAPVD1* | NM_015635 | 1578 | 114080 | 3 | 178 | 0.000560 | 0.981050 | 1 |
| *ITGA3* | NM_002204 | 2262 | 108745 | 1 | 178 | 1.336480 | 0.247660 | 1 |
| *ITGB4* | NM_000213 | 4829 | 113954 | 2 | 178 | 3.518130 | 0.060700 | 1 |
| *ITSN1* | NM_001001132 | 1737 | 110486 | 1 | 178 | 0.610940 | 0.434430 | 1 |
| *ITSN2* | NM_006277 | 2486 | 113522 | 6 | 178 | 0.670860 | 0.412750 | 1 |
| *KANK1* | NM_001256877 | 4048 | 115160 | 3 | 178 | 1.257310 | 0.262160 | 1 |
| *KANK2* | NM_001136191 | 2120 | 112463 | 2 | 178 | 0.221650 | 0.637790 | 1 |
| *KANK4* | NM_181712 | 2063 | 115941 | 4 | 178 | 0.035360 | 0.850840 | 1 |
| *KIRREL* | NM_018240 | 1255 | 110481 | 3 | 178 | 0.113650 | 0.736030 | 1 |
| *LAMA5* | NM_005560 | 13382 | 105987 | 21 | 178 | 0.048200 | 0.826220 | 1 |
| *LAMB2* | NM_002292 | 4422 | 115802 | 2 | 178 | 2.822050 | 0.092980 | 1 |
| *LCAT* | NM_000229.2 | 453 | 108175 | 0 | 178 | 0.080588 | 0.776502 | 1 |
| *MAGI2* | NM_012301 | 2188 | 111282 | 2 | 178 | 0.290600 | 0.589830 | 1 |
| ***MMACHC*** | **NM_015506** | **1355** | **116227** | **9** | **178** | **19.844700** | **0.000010** | **0.00059** |
| *MYO1E* | NM_004998 | 2803 | 113227 | 1 | 178 | 1.963940 | 0.161090 | 1 |
| *NEU1* | NM_000434.4 | 315 | 113450 | 0 | 178 | 0.000087 | 0.992549 | 1 |
| *NPHS1* | NM_004646 | 2362 | 113019 | 3 | 178 | 0.013180 | 0.908590 | 1 |
| *NPHS2* | NM_014625 | 661 | 106398 | 1 | 178 | 0.141770 | 0.706530 | 1 |
| *NPHP4* | NM_015102 | 4147 | 111911 | 8 | 178 | 0.128180 | 0.720330 | 1 |
| *NUP107* | NM_020401 | 1480 | 114004 | 4 | 178 | 0.617560 | 0.431950 | 1 |
| *NUP133* | NM_018230 | 1601 | 112822 | 3 | 178 | 0.000290 | 0.986510 | 1 |
| *NUP160* | NM_015231 | 2624 | 114180 | 1 | 178 | 1.677590 | 0.195240 | 1 |
| *NUP205* | NM_015135 | 2975 | 115076 | 2 | 178 | 0.983980 | 0.321220 | 1 |
| *NUP85* | NM_024844.5 | 609 | 113732 | 0 | 178 | 0.215838 | 0.642229 | 1 |
| *NUP93* | NM_014669.5 | 1118 | 112201 | 0 | 178 | 0.922630 | 0.336780 | 1 |
| *OSGEP* | NM_017807 | 465 | 112630 | 0 | 178 | 0.074877 | 0.784363 | 1 |
| *PDSS2* | NM_020381 | 724 | 111025 | 1 | 178 | 0.100140 | 0.751660 | 1 |
| *PLCE1* | NM_016341 | 4112 | 115395 | 1 | 178 | 3.831910 | 0.050290 | 1 |
| *PMM2* | NM_000303 | 659 | 110988 | 3 | 178 | 1.971110 | 0.160330 | 1 |
| *PTPRO* | NM_030667 | 1362 | 115171 | 2 | 178 | 0.075190 | 0.783930 | 1 |
| *SCARB2* | NM_005506 | 1001 | 116094 | 1 | 178 | 0.000760 | 0.978020 | 1 |
| *SGPL1* | NM_003901 | 734 | 112888 | 2 | 178 | 0.101360 | 0.750210 | 1 |
| *SMARCAL1* | NM_014140 | 1445 | 114627 | 6 | 178 | 4.763400 | **0.029070** | 1 |
| *TNS2* | NM_015319 | 2576 | 110875 | 3 | 178 | 0.099630 | 0.752280 | 1 |
| *TP53RK* | NM_033550.4 | 250 | 96476 | 0 | 178 | 0.003420 | 0.953365 | 1 |
| *TPRKB* | NM_001330386 | 115 | 106906 | 0 | 178 | 0.500345 | 0.479349 | 1 |
| *TTC21B* | NM_024753 | 2630 | 115229 | 3 | 178 | 0.079450 | 0.778050 | 1 |
| *WDR73* | NM_032856 | 492 | 107953 | 0 | 178 | 0.119325 | 0.729768 | 1 |
| *XPO5* | NM_020750 | 2038 | 112074 | 1 | 178 | 0.947870 | 0.330260 | 1 |
| *ZMPSTE24* | NM_005857 | 680 | 115503 | 1 | 178 | 0.196550 | 0.657520 | 1 |

| **Supplementary Table 3:** Comparison of rare heterozygous variants (independent of pathogenicity predictions) between gnomAD and confirmed monogenic SRNS patients | | | | | | | |  |
| --- | --- | --- | --- | --- | --- | --- | --- | --- |
|  |  | **gnomAD** | | **Patients (confirmed monogenic n=41)** | |  |  |  |
| Gene | Accession # | AC (HET) | AN | AC (HET) | AN | Chi-2 | p-value | P-value with Bonferroni correction |
| *ADCK4* | NM_024876 | 943 | 114018 | 1 | 82 | 0.047350 | 0.827740 | 1 |
| *ALG1* | NM_019109 | 1284 | 110804 | 1 | 82 | 0.216000 | 0.642100 | 1 |
| *ANKFY1* | NM_001257999 | 1720 | 111605 | 0 | 82 | 0.468336 | 0.493754 | 1 |
| *ARHGDIA* | NM_001185077.1 | 368 | 86011 | 0 | 82 | 0.064099 | 0.800131 | 1 |
| *AVIL* | NM_006576 | 1495 | 115537 | 0 | 82 | 0.300168 | 0.583777 | 1 |
| *CD151* | NM_004357 | 528 | 115000 | 0 | 82 | 0.040943 | 0.839649 | 1 |
| *CD2AP* | NM_012120 | 991 | 115634 | 0 | 82 | 0.058795 | 0.808410 | 1 |
| *CDK20* | NM_001039803.2 | 732 | 109062 | 0 | 82 | 0.004571 | 0.946095 | 1 |
| *CFH* | NM_000186 | 1543 | 114823 | 0 | 82 | 0.332878 | 0.563969 | 1 |
| *COL4A3* | NM_000091 | 2801 | 112570 | 2 | 82 | 0.106280 | 0.744420 | 1 |
| *COL4A4* | NM_000092 | 2359 | 115038 | 2 | 82 | 0.020060 | 0.887360 | 1 |
| *COQ2* | NM_001358921.1 | 654 | 89426 | 1 | 82 | 0.016820 | 0.896800 | 1 |
| *COQ6* | NM_182480.2 | 1176 | 111596 | 0 | 82 | 0.154750 | 0.694037 | 1 |
| *CRB2* | NM_173689 | 2198 | 99200 | 2 | 82 | 0.056620 | 0.811920 | 1 |
| *CUBN* | NM_001081 | 7434 | 116016 | 3 | 82 | 0.625330 | 0.429070 | 1 |
| *DGKE* | NM_003647 | 1297 | 112590 | 0 | 82 | 0.211362 | 0.645702 | 1 |
| *DLC1* | NM_182643 | 3586 | 114447 | 3 | 82 | 0.001950 | 0.964780 | 1 |
| *EMP2* | NM_001424 | 635 | 116385 | 0 | 82 | 0.006303 | 0.936721 | 1 |
| *FAT1* | NM_005245 | 9183 | 115311 | 2 | 82 | 2.701370 | 0.100260 | 1 |
| *GAPVD1* | NM_015635 | 1578 | 114080 | 1 | 82 | 0.119750 | 0.729310 | 1 |
| *ITGA3* | NM_002204 | 2262 | 108745 | 2 | 82 | 0.025400 | 0.873370 | 1 |
| *ITGB4* | NM_000213 | 4829 | 113954 | 1 | 82 | 1.171370 | 0.279120 | 1 |
| *ITSN1* | NM_001001132 | 1737 | 110486 | 0 | 82 | 0.490333 | 0.483779 | 1 |
| *ITSN2* | NM_006277 | 2486 | 113522 | 3 | 82 | 0.281790 | 0.595530 | 1 |
| *KANK1* | NM_001256877 | 4048 | 115160 | 1 | 82 | 0.686610 | 0.407320 | 1 |
| *KANK2* | NM_001136191 | 2120 | 112463 | 1 | 82 | 0.001360 | 0.970600 | 1 |
| *KANK4* | NM_181712 | 2063 | 115941 | 1 | 82 | 0.001190 | 0.972490 | 1 |
| *KIRREL1* | NM_018240 | 1255 | 110481 | 0 | 82 | 0.201812 | 0.653262 | 1 |
| *LAMA5* | NM_005560 | 13382 | 105987 | 1 | 82 | 8.662490 | **0.003250** | 0.19175 |
| *LAMB2* | NM_002292 | 4422 | 115802 | 0 | 82 | 2.298183 | 0.129525 | 1 |
| *LCAT* | NM_000229.2 | 453 | 108175 | 0 | 82 | 0.072075 | 0.788338 | 1 |
| *MAGI2* | NM_012301 | 2188 | 111282 | 0 | 82 | 0.782185 | 0.376474 | 1 |
| *MMACHC* | NM_015506 | 1355 | 116227 | 0 | 82 | 0.219711 | 0.639260 | 1 |
| *MYO1E* | NM_004998 | 2803 | 113227 | 1 | 82 | 0.141620 | 0.706680 | 1 |
| *NEU1* | NM_000434.4 | 315 | 113450 | 0 | 82 | 0.327493 | 0.567139 | 1 |
| *NPHS1* | NM_004646 | 2362 | 113019 | 0 | 82 | 0.877420 | 0.348909 | 1 |
| *NPHS2* | NM_014625 | 661 | 106398 | 0 | 82 | 0.000161 | 0.989861 | 1 |
| *NPHP4* | NM_015102 | 4147 | 111911 | 2 | 82 | 0.098962 | 0.753078 | 1 |
| *NUP107* | NM_020401 | 1480 | 114004 | 2 | 82 | 0.179948 | 0.671418 | 1 |
| *NUP133* | NM_018230 | 1601 | 112822 | 1 | 82 | 0.098792 | 0.753284 | 1 |
| *NUP160* | NM_015231 | 2624 | 114180 | 0 | 82 | 1.040494 | 0.307707 | 1 |
| *NUP205* | NM_015135 | 2975 | 115076 | 1 | 82 | 0.185805 | 0.666431 | 1 |
| *NUP85* | NM_024844.5 | 609 | 113732 | 0 | 82 | 0.008597 | 0.926125 | 1 |
| *NUP93* | NM_014669.5 | 1118 | 112201 | 2 | 82 | 0.574919 | 0.448311 | 1 |
| *OSGEP* | NM_017807 | 465 | 112630 | 0 | 82 | 0.077671 | 0.780478 | 1 |
| *PDSS2* | NM_020381 | 724 | 111025 | 1 | 82 | 0.002315 | 0.961622 | 1 |
| *PLCE1* | NM_016341 | 4112 | 115395 | 1 | 82 | 0.717045 | 0.397115 | 1 |
| *PMM2* | NM_000303 | 659 | 110988 | 1 | 82 | 0.000335 | 0.985390 | 1 |
| *PTPRO* | NM_030667 | 1362 | 115171 | 0 | 82 | 0.229901 | 0.631597 | 1 |
| *SCARB2* | NM_005506 | 1001 | 116094 | 0 | 82 | 0.060940 | 0.805016 | 1 |
| *SGPL1* | NM_003901 | 734 | 112888 | 1 | 82 | 0.002119 | 0.963281 | 1 |
| *SMARCAL1* | NM_014140 | 1445 | 114627 | 0 | 82 | 0.278692 | 0.597560 | 1 |
| *TNS2* | NM_015319 | 2576 | 110875 | 0 | 82 | 1.060434 | 0.303116 | 1 |
| *TP53RK* | NM_033550.4 | 250 | 96476 | 0 | 82 | 0.391186 | 0.531677 | 1 |
| *TPRKB* | NM_001330386 | 115 | 106906 | 0 | 82 | 1.928068 | 0.164970 | 1 |
| *TTC21B* | NM_024753 | 2630 | 115229 | 2 | 82 | 0.075582 | 0.783376 | 1 |
| *WDR73* | NM_032856 | 492 | 107953 | 0 | 82 | 0.043124 | 0.835492 | 1 |
| *XPO5* | NM_020750 | 2038 | 112074 | 0 | 82 | 0.670478 | 0.412885 | 1 |
| *ZMPSTE24* | NM_005857 | 680 | 115503 | 0 | 82 | 0.000645 | 0.979735 | 1 |

| **Supplementary Table 4:** Comparison of gnomAD and SRNS patients with no confirmed genetic diagnosis, for HPPV (low stringency prediction tools) | | | | | | | |  |
| --- | --- | --- | --- | --- | --- | --- | --- | --- |
|  |  | **gnomAD** | | **Patients (non-monogenic n=89)** | |  |  |  |
| Gene | Accession # | AC (HET) | AN | AC (HET) | AN | Chi-2 | p-value | p-value with Bonferroni correction |
| *ADCK4* | NM_024876 | 452 | 113416 | 0 | 178 | 0.061586 | 0.804006 | 1 |
| *ALG1* | NM_019109 | 303 | 111639 | 0 | 178 | 0.000649 | 0.979674 | 1 |
| *ANKFY1* | NM_001257999 | 436 | 112196 | 1 | 178 | 0.053664 | 0.816807 | 1 |
| *ARHGDIA* | NM_001185077.1 | 40 | 98923 | 0 | 178 | 2.557131 | 0.109798 | 1 |
| *AVIL* | NM_006576 | 412 | 116176 | 0 | 178 | 0.027068 | 0.869318 | 1 |
| *CD151* | NM_004357 | 142 | 113622 | 0 | 178 | 0.348660 | 0.554873 | 1 |
| *CD2AP* | NM_012120 | 137 | 116653 | 0 | 178 | 0.407550 | 0.523216 | 1 |
| *CDK20* | NM_001039803.2 | 214 | 109842 | 0 | 178 | 0.068539 | 0.793476 | 1 |
| *CFH* | NM_000186 | 222 | 113153 | 1 | 178 | 0.062583 | 0.802459 | 1 |
| *COL4A3* | NM_000091 | 1106 | 111971 | 0 | 178 | 0.908211 | 0.340590 | 1 |
| *COL4A4* | NM_000092 | 599 | 114094 | 0 | 178 | 0.202364 | 0.652820 | 1 |
| *COQ2* | NM_001358921.1 | 131 | 99333 | 0 | 178 | 0.302156 | 0.582534 | 1 |
| *COQ6* | NM_182480.2 | 164 | 113730 | 0 | 178 | 0.232484 | 0.629688 | 1 |
| *CRB2* | NM_173689 | 511 | 98785 | 0 | 178 | 0.192449 | 0.660886 | 1 |
| *CUBN* | NM_001081 | 2048 | 115729 | 2 | 178 | 0.151317 | 0.697279 | 1 |
| *DGKE* | NM_003647 | 132 | 112155 | 0 | 178 | 0.405519 | 0.524253 | 1 |
| *DLC1* | NM_182643 | 500 | 114210 | 0 | 178 | 0.099958 | 0.751880 | 1 |
| *EMP2* | NM_001424 | 113 | 114913 | 1 | 178 | 0.592145 | 0.441591 | 1 |
| *FAT1* | NM_005245 | 2244 | 113417 | 5 | 178 | 0.223052 | 0.636724 | 1 |
| *GAPVD1* | NM_015635 | 354 | 112438 | 0 | 178 | 0.006364 | 0.936416 | 1 |
| *ITGA3* | NM_002204 | 283 | 107782 | 0 | 178 | 0.002401 | 0.960919 | 1 |
| *ITGB4* | NM_000213 | 1217 | 113067 | 0 | 178 | 1.056589 | 0.303994 | 1 |
| *ITSN1* | NM_001001132 | 347 | 107703 | 0 | 178 | 0.009236 | 0.923440 | 1 |
| *ITSN2* | NM_006277 | 671 | 113808 | 0 | 178 | 0.288564 | 0.591142 | 1 |
| *KANK1* | NM_001256877 | 1110 | 115887 | 1 | 178 | 0.027032 | 0.869406 | 1 |
| *KANK2* | NM_001136191 | 276 | 112521 | 0 | 178 | 0.009457 | 0.922531 | 1 |
| *KANK4* | NM_181712 | 292 | 115614 | 0 | 178 | 0.005846 | 0.939051 | 1 |
| *KIRREL1* | NM_018240 | 187 | 106603 | 1 | 178 | 0.111500 | 0.738443 | 1 |
| *LAMA5* | NM_005560 | 2345 | 105937 | 6 | 178 | 0.526532 | 0.468069 | 1 |
| *LAMB2* | NM_002292 | 1186 | 115181 | 0 | 178 | 0.978172 | 0.322651 | 1 |
| *LCAT* | NM_000229.2 | 171 | 110173 | 0 | 178 | 0.182764 | 0.669009 | 1 |
| *MAGI2* | NM_012301 | 293 | 108785 | 0 | 178 | 0.000957 | 0.975318 | 1 |
| *MMACHC* | NM_015506 | 710 | 115837 | 0 | 178 | 0.321296 | 0.570830 | 1 |
| *MYO1E* | NM_004998 | 576 | 109701 | 0 | 178 | 0.202413 | 0.652780 | 1 |
| *NEU1* | NM_000434.4 | 109 | 113019 | 0 | 178 | 0.631571 | 0.426780 | 1 |
| *NPHS1* | NM_004646 | 440 | 112837 | 1 | 178 | 0.057025 | 0.811262 | 1 |
| *NPHS2* | NM_014625 | 314 | 113151 | 1 | 178 | 0.000012 | 0.997186 | 1 |
| *NPHP4* | NM_015102 | 755 | 111702 | 3 | 178 | 1.356711 | 0.244108 | 1 |
| *NUP107* | NM_020401 | 216 | 113637 | 1 | 178 | 0.074495 | 0.784901 | 1 |
| *NUP133* | NM_018230 | 232 | 113447 | 0 | 178 | 0.051496 | 0.820480 | 1 |
| *NUP160* | NM_015231 | 621 | 114238 | 0 | 178 | 0.226456 | 0.634164 | 1 |
| *NUP205* | NM_015135 | 612 | 113906 | 0 | 178 | 0.218201 | 0.640414 | 1 |
| *NUP85* | NM_024844.5 | 254 | 114742 | 0 | 178 | 0.028981 | 0.864823 | 1 |
| *NUP93* | NM_014669.5 | 132 | 110445 | 0 | 178 | 0.390534 | 0.532019 | 1 |
| *OSGEP* | NM_017807 | 85 | 114524 | 0 | 178 | 1.029536 | 0.310268 | 1 |
| *PDSS2* | NM_020381 | 95 | 112568 | 0 | 178 | 0.818812 | 0.365528 | 1 |
| *PLCe1* | NM_016341 | 1352 | 114222 | 1 | 178 | 0.183283 | 0.668567 | 1 |
| *PMM2* | NM_000303 | 373 | 111071 | 2 | 178 | 1.336542 | 0.247645 | 1 |
| *PTPRO* | NM_030667 | 270 | 115115 | 0 | 178 | 0.016650 | 0.897329 | 1 |
| *SCARB2* | NM_005506 | 112 | 115200 | 0 | 178 | 0.621199 | 0.430602 | 1 |
| *SGPL1* | NM_003901 | 63 | 112725 | 1 | 178 | 1.577537 | 0.209116 | 1 |
| *SMARCAL1* | NM_014140 | 126 | 112846 | 1 | 178 | 0.447806 | 0.503379 | 1 |
| *TNS2* | NM_015319 | 613 | 112548 | 0 | 178 | 0.227833 | 0.633136 | 1 |
| *TP53RK* | NM_033550.4 | 71 | 99759 | 0 | 178 | 1.106124 | 0.292926 | 1 |
| *TPRKB* | NM_001330386 | 48 | 105176 | 0 | 178 | 2.168450 | 0.140868 | 1 |
| *TTC21B* | NM_024753 | 940 | 115173 | 0 | 178 | 0.628968 | 0.427734 | 1 |
| *WDR73* | NM_032856 | 198 | 105774 | 0 | 178 | 0.084503 | 0.771285 | 1 |
| *XPO5* | NM_020750 | 159 | 111928 | 0 | 178 | 0.243452 | 0.621724 | 1 |
| *ZMPSTE24* | NM_005857 | 247 | 114842 | 1 | 178 | 0.035316 | 0.850936 | 1 |

| **Supplementary Table 5:** Comparison of HPPV between gnomAD and confirmed monogenic SRNS patients (low stringency prediction tools) | | | | | | | |  |
| --- | --- | --- | --- | --- | --- | --- | --- | --- |
|  |  | **gnomAD** | | **Patients (monogenic n=41)** | |  |  |  |
| Gene | Accession # | AC (HET) | AN | AC (HET) | AN | Chi-2 | p-value | p-value with Bonferroni correction |
| *ADCK4* | NM_024876 | 452 | 113416 | 0 | 82 | 0.092552 | 0.760958 | 1 |
| *ALG1* | NM_019109 | 303 | 111639 | 0 | 82 | 0.347722 | 0.555405 | 1 |
| *ANKFY1* | NM_001257999 | 436 | 112196 | 0 | 82 | 0.104020 | 0.747057 | 1 |
| *ARHGDIA* | NM_001185077.1 | 40 | 98923 | 0 | 82 | 6.587339 | **0.010271** | 0.605989 |
| *AVIL* | NM_006576 | 412 | 116176 | 0 | 82 | 0.151540 | 0.697068 | 1 |
| *CD151* | NM_004357 | 142 | 113622 | 0 | 82 | 1.546703 | 0.213623 | 1 |
| *CD2AP* | NM_012120 | 137 | 116653 | 0 | 82 | 1.697216 | 0.192653 | 1 |
| *CDK20* | NM_001039803.2 | 214 | 109842 | 0 | 82 | 0.727641 | 0.393649 | 1 |
| *CFH* | NM_000186 | 222 | 113153 | 0 | 82 | 0.717767 | 0.396877 | 1 |
| *COL4A3* | NM_000091 | 1106 | 111971 | 1 | 82 | 0.127732 | 0.720795 | 1 |
| *COL4A4* | NM_000092 | 599 | 114094 | 0 | 82 | 0.011395 | 0.914989 | 1 |
| *COQ2* | NM_001358921.1 | 131 | 99333 | 0 | 82 | 1.424807 | 0.232614 | 1 |
| *COQ6* | NM_182480.2 | 164 | 113730 | 0 | 82 | 1.236617 | 0.266124 | 1 |
| *CRB2* | NM_173689 | 511 | 98785 | 0 | 82 | 0.013776 | 0.906568 | 1 |
| *CUBN* | NM_001081 | 2048 | 115729 | 2 | 82 | 0.000121 | 0.991224 | 1 |
| *DGKE* | NM_003647 | 132 | 112155 | 0 | 82 | 1.691986 | 0.193339 | 1 |
| *DLC1* | NM_182643 | 500 | 114210 | 0 | 82 | 0.055919 | 0.813067 | 1 |
| *EMP2* | NM_001424 | 113 | 113913 | 0 | 82 | 2.160605 | 0.141589 | 1 |
| *FAT1* | NM_005245 | 2244 | 113417 | 0 | 82 | 0.791646 | 0.373603 | 1 |
| *GAPVD1* | NM_015635 | 354 | 112438 | 0 | 82 | 0.227925 | 0.633067 | 1 |
| *ITGA3* | NM_002204 | 283 | 107782 | 0 | 82 | 0.378445 | 0.538436 | 1 |
| *ITGB4* | NM_000213 | 1217 | 113067 | 1 | 82 | 0.177412 | 0.673607 | 1 |
| *ITSN1* | NM_001001132 | 347 | 107703 | 0 | 82 | 0.211840 | 0.645329 | 1 |
| *ITSN2* | NM_006277 | 671 | 113808 | 0 | 82 | 0.000594 | 0.980554 | 1 |
| *KANK1* | NM_001256877 | 1110 | 115887 | 1 | 82 | 0.112038 | 0.737836 | 1 |
| *KANK2* | NM_001136191 | 276 | 112521 | 0 | 82 | 0.446252 | 0.504120 | 1 |
| *KANK4* | NM_181712 | 292 | 115614 | 1 | 82 | 0.412861 | 0.520521 | 1 |
| *KIRREL1* | NM_018240 | 187 | 106603 | 0 | 82 | 0.885312 | 0.346751 | 1 |
| *LAMA5* | NM_005560 | 2345 | 105937 | 1 | 82 | 0.063912 | 0.800417 | 1 |
| *LAMB2* | NM_002292 | 1186 | 115181 | 0 | 82 | 0.141597 | 0.706699 | 1 |
| *LCAT* | NM_000229.2 | 171 | 110173 | 0 | 82 | 1.095436 | 0.295270 | 1 |
| *MAGI2* | NM_012301 | 293 | 108785 | 0 | 82 | 0.354713 | 0.551457 | 1 |
| *MMACHC* | NM_015506 | 710 | 115837 | 0 | 82 | 0.000010 | 0.997460 | 1 |
| *MYO1E* | NM_004998 | 576 | 109701 | 0 | 82 | 0.011382 | 0.915038 | 1 |
| *NEU1* | NM_000434.4 | 109 | 113019 | 0 | 82 | 2.246297 | 0.133935 | 1 |
| *NPHS1* | NM_004646 | 440 | 112837 | 0 | 82 | 0.102415 | 0.748950 | 1 |
| *NPHS2* | NM_014625 | 314 | 113151 | 0 | 82 | 0.327971 | 0.566856 | 1 |
| *NPHP4* | NM_015102 | 755 | 111702 | 0 | 82 | 0.005273 | 0.942114 | 1 |
| *NUP107* | NM_020401 | 216 | 113637 | 0 | 82 | 0.762864 | 0.382434 | 1 |
| *NUP133* | NM_018230 | 232 | 113447 | 0 | 82 | 0.661316 | 0.416096 | 1 |
| *NUP160* | NM_015231 | 621 | 114238 | 0 | 82 | 0.006726 | 0.934638 | 1 |
| *NUP205* | NM_015135 | 612 | 113906 | 0 | 82 | 0.008156 | 0.928038 | 1 |
| *NUP85* | NM_024844.5 | 254 | 114742 | 0 | 82 | 0.561273 | 0.453748 | 1 |
| *NUP93* | NM_014669.5 | 132 | 110445 | 0 | 82 | 1.653960 | 0.198421 | 1 |
| *OSGEP* | NM_017807 | 85 | 114524 | 0 | 82 | 3.176121 | 0.074722 | 1 |
| *PDSS2* | NM_020381 | 95 | 112568 | 0 | 82 | 2.688572 | 0.101070 | 1 |
| *PLCe1* | NM_016341 | 1352 | 114222 | 1 | 82 | 0.242845 | 0.622159 | 1 |
| *PMM2* | NM_000303 | 373 | 111071 | 0 | 82 | 0.184453 | 0.667574 | 1 |
| *PTPRO* | NM_030667 | 270 | 115115 | 0 | 82 | 0.494482 | 0.481935 | 1 |
| *SCARB2* | NM_005506 | 112 | 115200 | 0 | 82 | 2.221511 | 0.136100 | 1 |
| *SGPL1* | NM_003901 | 63 | 112725 | 0 | 82 | 4.510699 | **0.033683** | 1 |
| *SMARCAL1* | NM_014140 | 126 | 112846 | 0 | 82 | 1.827329 | 0.176443 | 1 |
| *TNS2* | NM_015319 | 613 | 112548 | 0 | 82 | 0.006503 | 0.935728 | 1 |
| *TP53RK* | NM_033550.4 | 71 | 99759 | 0 | 82 | 3.350663 | 0.067178 | 1 |
| *TPRKB* | NM_001330386 | 48 | 105176 | 0 | 82 | 5.730090 | **0.016677** | 0.983943 |
| *TTC21B* | NM_024753 | 940 | 115173 | 0 | 82 | 0.042975 | 0.835773 | 1 |
| *WDR73* | NM_032856 | 198 | 105774 | 0 | 82 | 0.785419 | 0.375489 | 1 |
| *XPO5* | NM_020750 | 159 | 111928 | 0 | 82 | 1.266879 | 0.260353 | 1 |
| *ZMPSTE24* | NM_005857 | 247 | 114842 | 0 | 82 | 0.596479 | 0.439924 | 1 |
